# Quantitative Muscle Ultrasonography Using 2D Textural Analysis: A Novel Approach to Assess Skeletal Muscle Structure and Quality in Chronic Kidney Disease

**DOI:** 10.1177/01617346211009788

**Published:** 2021-04-15

**Authors:** Thomas J. Wilkinson, Jed Ashman, Luke A. Baker, Emma L. Watson, Alice C. Smith

**Affiliations:** 1 Department of Health Sciences, University of Leicester, Leicester, UK; 2 Department of Cardiovascular Sciences, University of Leicester, Leicester, UK

**Keywords:** ultrasonography, textural analysis, skeletal muscle, chronic kidney disease

## Abstract

Chronic kidney disease (CKD) is characterized by progressive reductions in skeletal muscle function and size. The concept of muscle quality is increasingly being used to assess muscle health, although the best means of assessment remains unidentified. The use of muscle echogenicity is limited by an inability to be compared across devices. Gray level of co-occurrence matrix (GLCM), a form of image texture analysis, may provide a measure of muscle quality, robust to scanner settings. This study aimed to identify GLCM values from skeletal muscle images in CKD and investigate their association with physical performance and strength (a surrogate of muscle function). Transverse images of the rectus femoris muscle were obtained using B-mode 2D ultrasound imaging. Texture analysis (GLCM) was performed using ImageJ. Five different GLCM features were quantified: energy or angular second moment (ASM), entropy, homogeneity, or inverse difference moment (IDM), correlation, and contrast. Physical function and strength were assessed using tests of handgrip strength, sit to stand-60, gait speed, incremental shuttle walk test, and timed up-and-go. Correlation coefficients between GLCM indices were compared to each objective functional measure. A total of 90 CKD patients (age 64.6 (10.9) years, 44% male, eGFR 33.8 (15.7) mL/minutes/1.73 m^2^) were included. Better muscle function was largely associated with those values suggestive of greater image texture homogeneity (i.e., greater ASM, correlation, and IDM, lower entropy and contrast). Entropy showed the greatest association across all the functional assessments (*r* = −.177). All GLCM parameters, a form of higher-order texture analysis, were associated with muscle function, although the largest association as seen with image entropy. Image homogeneity likely indicates lower muscle infiltration of fat and fibrosis. Texture analysis may provide a novel indicator of muscle quality that is robust to changes in scanner settings. Further research is needed to substantiate our findings.

## Introduction

Skeletal muscle health decreases with advancing age and several disease states, including chronic kidney disease (CKD).^1,2^ This loss of skeletal muscle mass contributes to impairments in strength and physical function;^3^ however, these adverse changes cannot be entirely accounted for by changes in muscle size. The concept of muscle quality is increasingly being used to assess skeletal muscle health. However, its assessment is a challenging, yet important area of future research as it is not sufficiently defined for use in clinical practice.^4^ Muscle quality may be described as the capacity to generate force relative to the mass/volume of contractile tissue (i.e., its functionality),^5^ but it may also be thought of in terms of the observed architecture and composition of the muscle itself. Many methods used to quantify muscle quality are either invasive (e.g., muscle biopsies), costly and inaccessible (e.g., MRI), or may expose the individual to ionizing radiation (e.g., CT, DXA). Ultrasonography may provide healthcare professionals and researchers with a safe, non-invasive, low-cost means to measure and evaluate skeletal muscle and its morphologic changes (5). Ultrasound is a valid^6^ and recommended tool to monitor skeletal muscle health in CKD,^7^ used to accurately diagnose sarcopenia^8^ and predict mortality risk in CKD.^9^

The features extracted from ultrasound images commonly evaluate muscle size, for example, cross-sectional area (CSA) or muscle thickness.^3,5,9-12^ Ultrasound elastography^13,14^ and supersonic shear imaging^15^ can also be used to assess the mechanical and viscoelastic properties of tissue, including muscle stiffness, which contribute to physical function. However, these techniques are subject to variability and its unknown how reliable these measurements are in fibrotic muscle tissue, as found in CKD.^13^ Further, study has used beam-formed radio frequency (RF) to investigate dystrophic muscle^16^ and quantitative ultrasonography has revealed variations in attenuation related to the degree of fatty infiltration in boys with Duchenne muscular dystrophy (DMD) and other neuromuscular disorders.^16-18^ Muscle quality has also previously been assessed through the echogenicity, the characteristic of tissue, or other material to reflect ultrasonic waves. Measures of muscle echogenicity (or echo intensity, EI), through gray-scale analysis, is thought to represent infiltration of intramuscular adipose and fibrotic tissue.^5,19^ Echogenicity is associated with muscle function in older adults^20,21^ and our group has previously shown that EI is a strong predictor of physical function and exercise capacity in patients with CKD.^3^ Echo intensity provides a simple unidimensional analysis and classifies (the average) tissue as hyperechoic or hypoechoic. However, the *spatial* nature of the gray level distribution on an image is not considered with this approach.^22^ Echo intensity can be affected by the thickness of the tissue and is highly dependent on the ultrasound scanner settings—small differences in beam frequency or gain, or many other settings—can give completely different results^5,23,24^ making comparisons between machines, manufactures, or studies impossible. Thus, the use of EI analysis to estimate muscle quality is critically limited.^25^

In contrast to EI, higher-order (also known as second-order) texture features, that can be extracted from ultrasound images, are intensity invariant.^10,26,27^ Texture analysis is based on the spatial variation of the pixel intensity, and thus, considers, besides the pixels gray level variation, also the relative spatial position between pixels.^22^ One approach to texture analysis is the gray level of co-occurrence matrix (GLCM).^28^ A key advantage of GLCM, in comparison to conventional first-order features, such as EI, is that its parameters are robust to changes in ultrasonography scanner settings (i.e., gain or frequency).^25,26^ As such, higher-order and non-linear descriptors may offer better characterization performance than histogram-based parameters,^27,29^ and may provide a better reliable measure of muscle quality.

The literature regarding muscle texture analysis in human is still scarce^28^ although texture analysis of skeletal muscle ultrasound has potential clinical implications.^30^ Research has shown that texture analysis can distinguish the differences between muscle dynapenia and non-dynapenia,^31^ as well as identify differences between the muscle of those with muscle disorders and controls.^10,32,33^ Watanabe et al.^25^ reported that texture analysis parameters change with age, as well as being associated with muscle strength. However, further research is needed to better describe the use of texture analysis in skeletal muscle, and identify the parameters best associated with muscle function. To the best of our knowledge, in previous studies, only linear and first-order descriptors are used to characterize the texture of different skeletal muscles in CKD. This study aimed to perform texture analysis on skeletal muscle images in CKD and to (1) compare these values with muscle function and strength and (2) investigate demographic and clinical predictors of these parameters.

## Methods

### Participants

Patients, attending nephrology outpatient clinics between 2013 and 2020 at Leicester General Hospital, UK, were recruited if they had: CKD not requiring renal replacement therapy; aged ≥18; no significant co-morbidity (e.g., unstable hypertension, lethal arrhythmia) or physical impairment contraindicative to exercise; and sufficient ability to provide informed consent. This cross-sectional study is an exploratory analysis of pooled baseline skeletal muscle ultrasound data taken from two trials conducted by our group (ExTra-CKD: ISRCTN36489137 and DIMENSION-KD: ISRCTN84422148). These studies were approved by the East-Midlands Research Ethics Committee and conducted under the Declaration of Helsinki. All patients provided informed consent. Participant’s clinical details (kidney function by estimated glomerular filtration rate (eGFR), hemoglobin, albumin, and presence of hypertension and diabetes) were taken from their latest routine blood test. Body mass index (BMI) was recorded during the assessment visit.

### Ultrasound Image Acquisition

Transverse ultrasound images of the right rectus femoris were obtained with a B-mode 2D ultrasound imaging device (Hitachi EUB-6500). As previously described by work from our group^6,8^ and per procedures from the SARCUS working group on behalf of the Sarcopenia Special Interest Group of the European Geriatric Medicine Society, during the scan, patients were sat upright with knee flexion of ~120° and legs flat out in front. A linear transducer (7.5-MHz, 58-dB gain) was positioned perpendicular to the longitudinal axis of the quadriceps femoris at the midpoint between the greater trochanter and the proximal end of the patella.^5^ To ensure distortion and excess compression was avoided, the pressure was kept to a minimum and a generous amount of contact gel was applied. Three images were obtained (if all CSAs were within ±10% of each other; if not, a subsequent image was obtained and outliers discarded). Given that scan depth can influence texture analysis,^26^ scan depth (i.e., field of view) was kept at 55 mm.

### Muscle Quality

**Figure 1.**
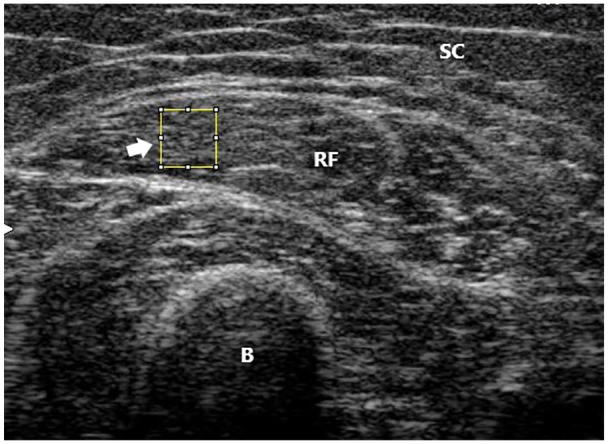
Texture analysis acquisition. SC = subcutaneous fat tissue; RF = rectus femoris muscle; B = bone (femur). Arrow donates region of interest (50 × 50 pixel square).

All image analysis was performed using ImageJ 1.52v (National Institutes of Health, USA). All images were visually inspected and analyzed by a single observer (JA), who was blinded to all personal information such as age and sex, as well as functional data. To assess muscle quality, first (i.e., EI)—and second (i.e., GLCM)-order statistical analyses were performed. Mean EI was determined using the distribution of the in-built histogram tool (0: black and 255: white). GLCM was assessed using a bespoke plugin macro (GLCM Texture v0.4, Julio Cabera). Before any analysis was performed, the image was transformed from RGB color to 8-bit. One equal-sized ROI (50 pixels × 50 pixels) was placed in the medial portion of the rectus femoris muscle (Figure 1). The dimension and the approximate position of the ROI was chosen to be the same for all patients to make the extracted features independent of ROI size.

#### Gray-level co-occurrence matrix (GLCM)

The GLCM is a mathematical method used for statistical 2D texture analysis proposed by Haralick et al.^34^ GLCM corresponds to a directional pattern counter with a specific distance *d* and angle *θ* between neighboring pixel pairs for the grayscale image. GLCM computation was performed in 4 directions: 0, 90, 180, and 270 with a distance parameter of 1. The corresponding displacement vectors are [0 1], [−1 1], [−1 0], and [−1 −1].^35^ In the proposed work, an averaged four-direction value was used for feature extraction to avoid dependence on the direction.^36^ Five different GLCM features were quantified: energy or angular second moment (ASM), entropy (ENT), homogeneity or inverse difference moment (IDM), correlation (COR), and contrast (CON):

- Angular second moment (ASM), also known as energy, maybe an indicator of grey level uniformity or homogeneity (i.e., similarity).^25^ When the image is homogeneous, the ASM will have a high value.^32^
- Entropy (ENT) is a measure of textural disorder within the analyzed structure.^25^ A homogeneous image will result in a lower entropy value.^32^
- Inverse difference moment (IDM) may be an indirect parameter of textural homogeneity (i.e., similarity) and is associated with pixel pairs.^25^ Higher values indicate a homogeneous image.^32^
- Correlation (COR) explains the relationship between a pixel and its neighbor over the whole image. Higher values can be obtained for similar gray-level regions.^32^
- Contrast (CON) is a measure of the intensity variation between a pixel and its neighbor over the whole image.^35^ The greater the variation (i.e., gray level dispersion) in an image, the greater the contrast.

**Figure 2.**
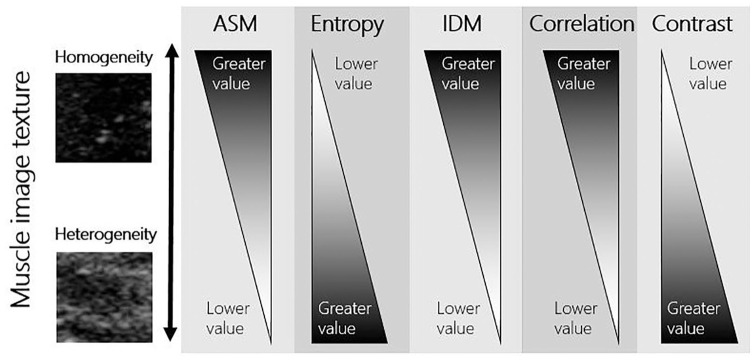
Image examples taken from scans of patients included in the current analysis—the image used as an example as high homogeneity was taken from the scan with the lowest entropy and highest ASM; the image used as an example as high heterogeneity was taken from the scan with the highest entropy and lowest ASM. ASM = energy or angular second moment; IDM = homogeneity or inverse difference moment.

These parameters summarize important information about the structural arrangement of surfaces by discerning likelihoods that pixels have the same or different gray-level values as their neighbors and distances between pixel pairs of equal intensity. A full description of the GLCM process can be found in Watanabe et al.^25^ Healthy skeletal muscle is largely homogenous in texture.^37^ A proposed schematic of how image texture (homo/heterogeneity) corresponds to these parameters can be found in Figure 2.

Full analysis was repeated for *n* = 5 patients to provide reliability data for the assessor (JA). No differences were seen for each GLCM parameter when re-assesed (*p* = .178 to .708) with intra-class correlation coeffiecnts between .277 and .951. Data can be found in Supplemental Material 1.

### Physical Function and Strength Tests

Physical function and strength were assessed using frequently used assessments previously described in Wilkinson et al.^1^ Handgrip strength (HGS) of the dominant and non-dominant hands were assessed using a JAMAR hydraulic handheld dynamometer (Fabrication Enterprises Inc, Irvington, New York). Each participant had three attempts with each hand and the maximal score was taken for both. The sit to stand-60 (STS-60) test was employed as measures of lower body strength and muscle endurance. For this test, the patient sat on a seat (17 in. [43.2 cm] from the ground) with their feet slightly apart. With their hands across their chest, patients were asked to perform as many STS cycles in 60 seconds. Usual gait speed was measured over a marked 4 m course, with the faster of two trials used for analysis. Validated in CKD,^38^ the incremental shuttle walk test (ISWT) was used as a measure of exercise capacity and cardiorespiratory fitness. For this test, the patient was required to walk between two cones 10 m apart. Patients maintained a speed regulated by an external auditory beep. The walking speed was initially very slow (0.5 m/second), but for every minute stage, the required walking speed increased by 0.2 m/second. The patient maintained cadence with the beeps until volitional exhaustion or until they could no longer keep up with the required pace. The total number of shuttles and distance walked (m) was calculated.

### Statistical Analysis

Unless stated, all data are shown as mean and standard deviation. Patterns of data missingness were checked by Little’s MCAR and separate variance *T* tests, and to maximize the sample size, missing data (variables detailed in Table 1) was imputed using an appropriate method (Expectation-Maximization^39^ procedure, using all variables available as predictors) in IBM SPSS, v26. This method introduces less bias than more conservative approaches (e.g., mean substitution). Given the high multicollinearity between the GLCM parameters, they could not be entered into a linear regression model simultaneously. As such, to identify the GLCM parameters predictive of physical function and strength, we used Spearman’s ρ correlation co-efficient as variables were non-normally distributed. Significant variables identified were displayed visually by plotting the GLCM parameter against the physical function test in a scatter plot diagram.

**Table 1.** Patient Characteristics.

*N* = 90	
Age, years^†^	64.6 (10.9)
Sex, males *n* (%)	40 (44)
Ethnicity, *n* (%)	
White	63 (70)
South Asian	8 (9)
Other	1 (1)
BMI, kg/m^2†^	29.2 (6.0)
eGFR, mL/minutes/1.73 m^2†^	33.8 (15.7)
Hemoglobin, g/L^†^	125.7 (15.9)
Albumin, g/L^†^	42.1 (2.5)
Hypertension, *n* (%)	50 (55)
Diabetes, *n* (%)	17 (19)
Texture analysis^†^	
ASM, AU	0.002 (0.002)
Contrast, AU	607.9 (152.7)
Correlation, AU	0.004 (0.002)
IDM, AU	0.36 (0.78)
Entropy, AU	25.1 (0.8)
Echo intensity, AU	59.3 (21.1)
Physical function and strength^†^	
ISWT, meters walked	433.0 (178.7)
Gait speed, m/second	1.3 (0.4)
Handgrip strength, kg	28.9 (10.2)
TUAG, seconds	9.7 (2.4)
STS-60, repetitions	25.8 (10.4)

Unless stated otherwise, data shown as mean (standard deviation).

BMI = body mass index; eGFR = estimated glomerular filtration rate; AU = arbitrary units; ASM = energy or angular second moment; IDM = homogeneity or inverse difference moment; ISWT = incremental shuttle walk test; TUAG = time up-and-go test.

† Imputed data.

To quantify the best overall GLCM parameter, correlation coefficients (*r*) between each of the GLCM indices were averaged for each objective functional measure, for example, each ρ value between the entropy and the rest of the objective tests (i.e., HGS, STS-60, gait speed, TUAG, ISWT) were averaged to determine a mean *r* for entropy. To reduce skew in the *r* distribution^40^ *r* values were first transformed into normally distributed Fisher-*ɀ* values (ɀ′). Following conversion to *ɀ*′, these values were averaged (as above) before the mean ɀ0 value was back-converted to a final *r* value (*rɀ*′) using the inverse formula. To explore demographic and clinical predictors of GLCM parameters, the following variables were entered into a general linear model: age, sex, ethnicity, eGFR, Hb, albumin, and BMI. We used bivariate correlations to explore the relationship between individual GLCM parameters and EI. Significance was set at *p* < .050. Data were analyzed using IBM SPSS Statistics 26 and GraphPad Prism 8, and are presented as means (*SD*) unless otherwise stated.

## Results

### Participant Characteristics

Participant demographic and clinical characteristics can be found in Table 1. Overall, the mean age of participants was 64.6 (10.9) years with 44% of the cohort male and the majority (70%) from White ethnic background. Clinically, participants were largely overweight (BMI of 29.2 (6.0) kg/m^2^) and had mild CKD (eGFR of 33.8 (15.7) mL/minutes/1.73 m^2^, albumin of 42.1 (2.5) g/L). The majority of patients were comorbid with a hypertension prevalence of 55%.

### Association of GLCM Parameters and Echo Intensity

**Figure 3.**
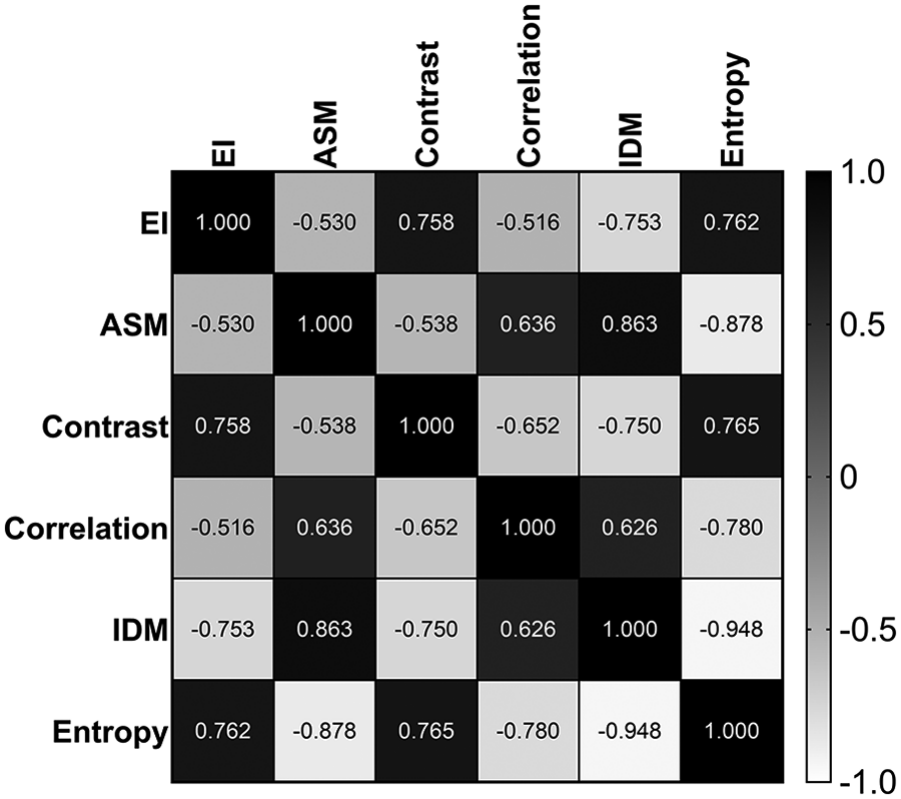
Correlation matrix between gray-level co-occurrence matrix (GLCM) parameters and echo intensity. EI = echo intensity; ASM = energy or angular second moment; IDM = homogeneity or inverse difference moment.

Greater EI (more fat infiltration) taken from the ROI was significantly associated with a lower ASM (i.e., low homogeneous image texture) (*r* = −.530, *p* < .001), lower correlation (*r* = −.516, *p* < .001), and lower IDM (i.e., low homogeneous) (*r* = −.753, *p* < .001). Greater EI (more fat infiltration) was associated with a higher contrast (i.e., higher gray level dispersion) (*r* = .758, *p* < .001) and higher entropy (i.e., higher heterogeneous texture) (*r* = .762, *p* < .001). A figure depicting the relationship between GLCM parameters and EI can be found in Figure 3.

### Association of GLCM Parameters and Physical Function

**Figure 4.**
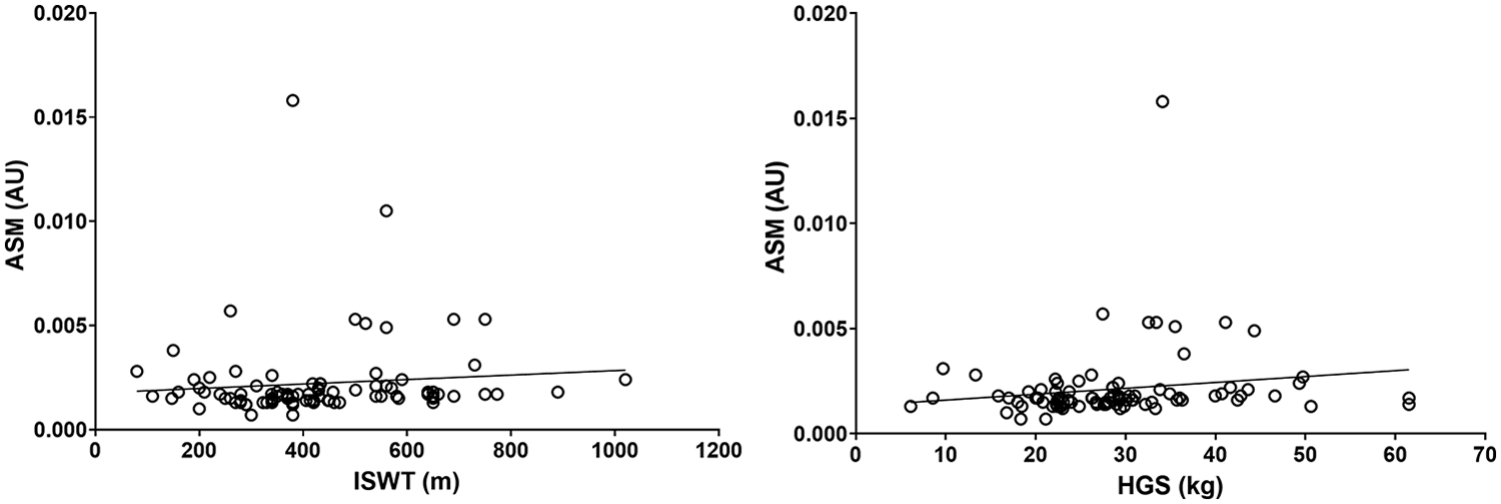
Association between angular second moment (ASM) and physical function (ASM plotted against incremental shuttle walk test (ISWT); and ASM plotted against handgrip strength (HGS)).

**Figure 5.**
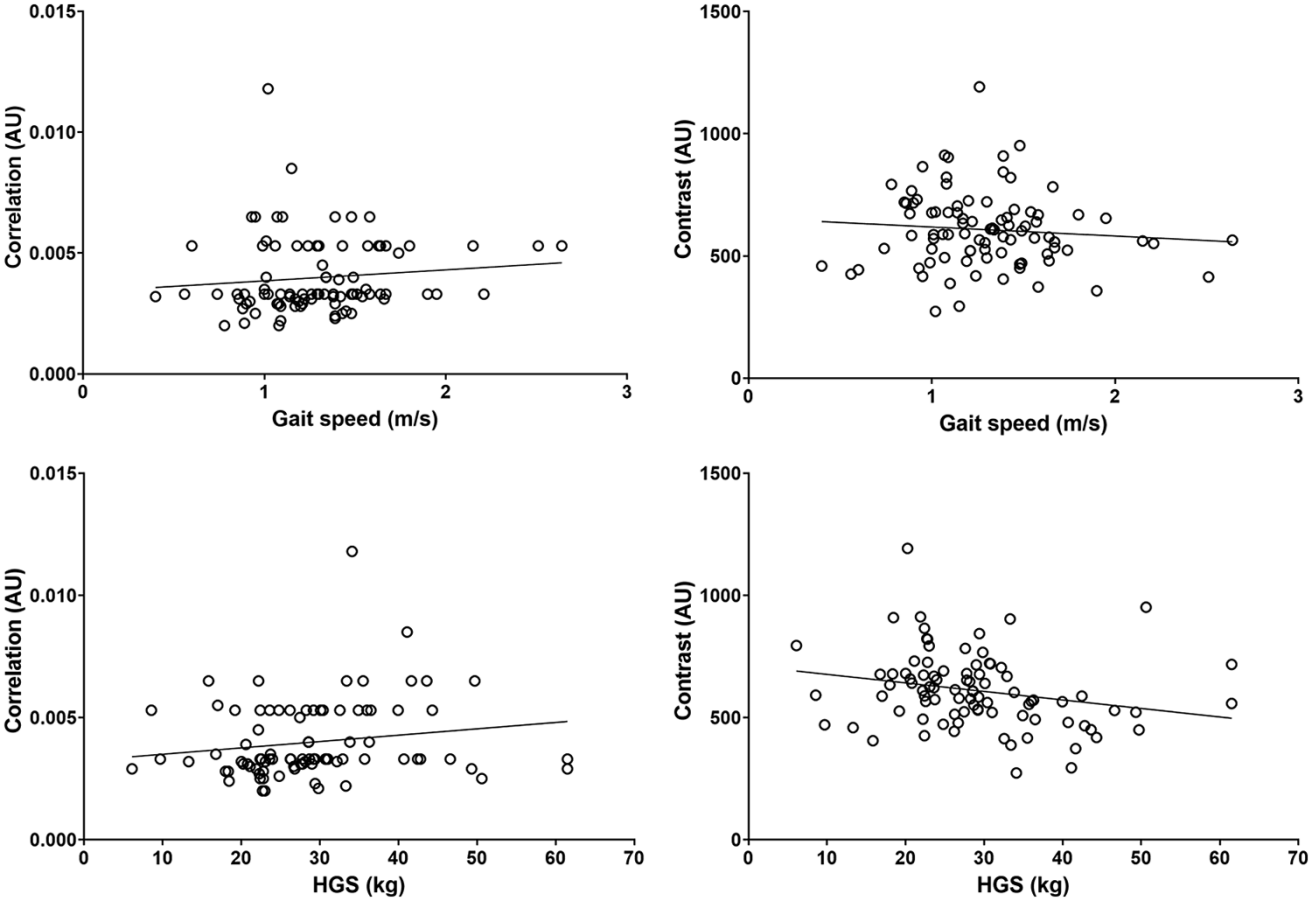
Association between correlation and contract and physical function. HGS = handgrip strength.

**Figure 6.**
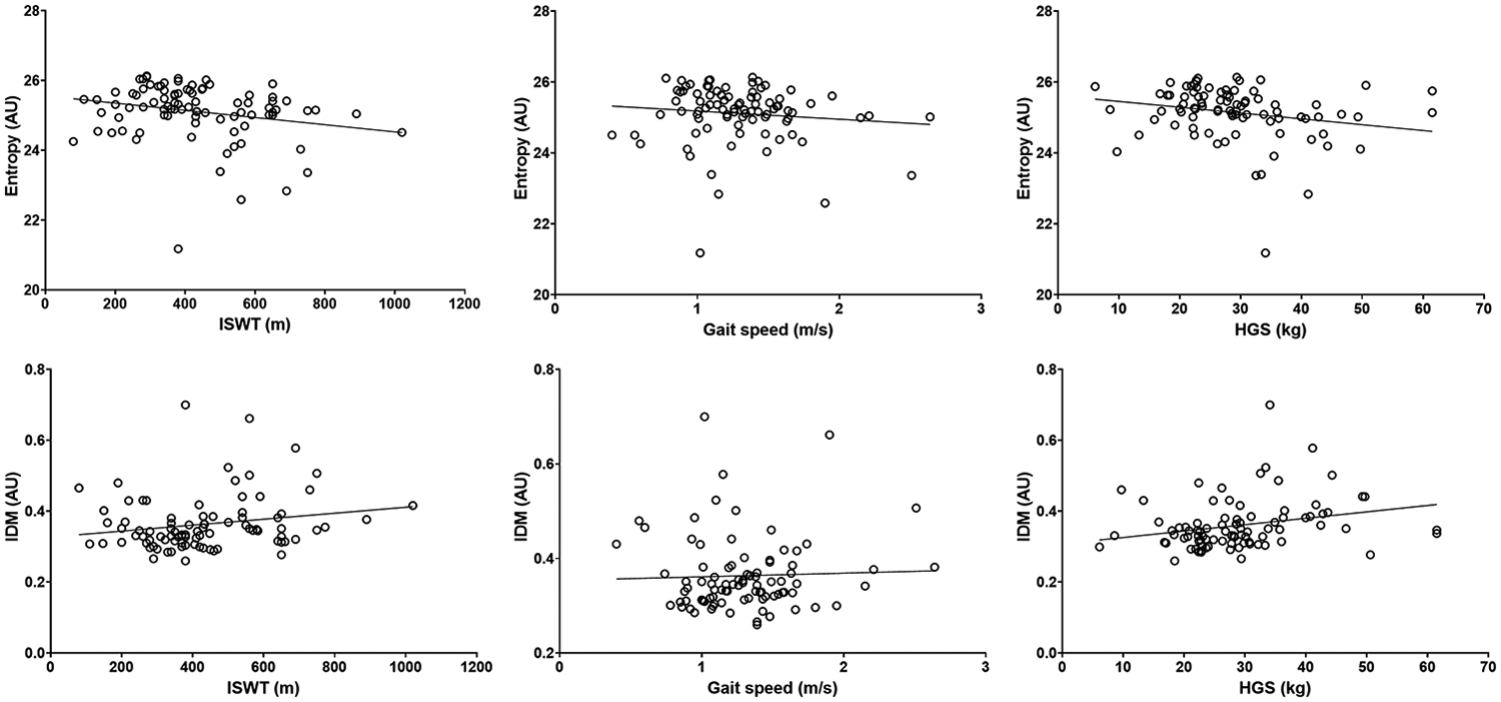
Association between entropy and IDM and physical function. IDM = homogeneity or inverse difference moment; ISWT = incremental shuttle walk test (ISWT); HGS = handgrip strength.

The mean GLCM and physical function values can be found in Table 1. GLCM parameters explained 20% of the variance in the ISWT, 7% of gait speed, 8% of HGS, 11% of the TUAG, and 14% of the STS-60. Overall, greater image texture homogeneity was associated with better physical function and greater strength, although whilst significant, associations were weak (Table 2 and Figures 4–6). Higher ASM was significantly associated with a greater distance walked on the ISWT (ρ = .215, *p* = .042) and a higher maximum HGS (ρ = .235, *p* = .026). Lower entropy was associated with greater distance walked on the ISWT (ρ = −.259, *p* = .014) and a higher maximum HGS (ρ = −.255, *p* = .015), as well as a quicker gait speed (ρ = −.275, *p* = .009). Greater performance on the ISWT, HGS, and gait speed tests were also associated with a higher IDM (ρ = .216, *p* = .041; ρ = .289, *p* = .006; ρ = .244, *p* = .021, respectively). Greater correlation was associated with a quicker gait speed (ρ = .289, *p* = .006) and higher HGS (ρ = .244, *p* = .021). Conversely, better performance on these tests was associated with lower contrast (ρ = −.255, *p* = .015; ρ = −.275, *p* = .009). No associations were seen between any GLCM parameters and the TAUG and STS-60 test. The average co-efficient for each GLCM parameter can be found in Table 2. Entropy showed the greatest association across all the functional assessments (*r* = −.177).

**Table 2.** Association between Texture Analysis Parameters and Physical Function and Strength.

*n* = 90	ISWT		Gait speed		TUAG		STS-60		HGS		Average co-efficient (*r*)
	ρ	*p*	ρ	*p*	ρ	*p*	ρ	*p*	ρ	*p*	
ASM	.215	.042*	.188	.077	**−**.104	.330	.110	.303	.235	.026*	0.130
Entropy	−.259	.014*	−.275	.009*	.079	.461	**−**.163	.126	−.255	.015*	−0.177^†^
IDM	.216	.041*	.244	.021*	**−**.053	.621	.129	.227	.289	.006*	0.167
Correlation	.141	.185	.289	.006*	**−**.037	.731	.106	.318	.244	.021*	0.150
Contrast	**−**.166	.119	−.255	.015*	.107	.317	**−**.136	.200	−.275	.009*	–0.147

Analyzed using Spearman’s ρ.

ASM = energy or angular second moment; IDM = homogeneity or inverse difference moment; ISWT = incremental shuttle walk test; TUAG = time up-and-go test; HGS = handgrip strength.

† Largest average coefficient following Fisher transformation. *Statistical significance recognised as *p* < .050.

### Predictors of GLCM Parameters

Table 3 shows the demographic and clinical predictors of the GLCM parameters. Significant different between sexes were seen for correlation and contrast. Hb associated with greater ASM, IDM, and lower entropy (more homogeneity in image texture). Higher albumin was associated with reduced ASM, whilst increased BMI was associated with greater correlation values. We observed no association between age, ethnicity, or eGFR on texture parameters.

**Table 3.** Predictors of Texture Analysis Parameters.

	ASM			Entropy			IDM			Correlation			Contrast		
	β	*t*	*p*	β	*t*	*p*	β	*t*	*p*	β	*t*	*p*	β	*t*	*p*
Age	.024	.228	.820	−.052	−.512	.610	.103	1.003	.319	−.059	−.576	.566	−.077	−.762	.448
Sex	−.028	−.230	.819	.194	1.654	.102	−.097	−.816	.417	−.287	−2.434	.017*	.305	2.620	.010*
Ethnicity	−.098	−.901	.370	.031	.296	.768	.033	.311	.757	−.072	−.677	.500	.013	.127	.899
eGFR	−.157	−1.180	.241	.251	1.963	.053	−.189	−1.456	.149	−.150	−1.166	.247	.229	1.797	.076
Hb	.389	2.387	.019*	−.323	−2.057	.043*	.394	2.467	.016*	−.049	−.312	.756	−.152	−.971	.334
Albumin	−.244	−2.068	.042*	.104	.909	.366	−.139	−1.204	.232	.107	.935	.352	.028	.246	.806
BMI	.066	.581	.563	−.152	−1.385	.170	.113	1.017	.312	.312	2.820	.006*	−.182	−1.669	.099

ASM = energy or angular second moment; IDM = homogeneity or inverse difference moment; eGFR = estimated glomerular filtration rate; Hb = hemoglobin; BMI = body mass index.

* Statisical signifcance recognised as *p* < .050.

## Discussion

### Summary of Findings

Using novel GLCM texture analysis of skeletal muscle ultrasound images, we found that better muscle function was largely associated with those values suggestive of greater image texture homogeneity (i.e., greater ASM, correlation, and IDM, lower entropy and contrast). Lower entropy may be the best indicator of muscle function, and therefore muscle quality. Greater texture homogeneity is likely indicative of a reduced presence of fibrous and adipose tissue in the muscles. However, the relationships between function and GLCM parameters were moderate with GLCM variables explaining between 7% and 20% of the variance in functional tests; this suggests other factors may be of importance.

### Interpretation of Findings

Given its relative novelty and its limited use, the role of GLCM and texture analysis in determining skeletal muscle quality in CKD is unknown. This analysis provides the first investigation of the use of ultrasound-derived GLCM in CKD muscle. We decided to explore the association with muscle function to ascertain what GLCM parameters have the most useful clinical utility. Such parameters may indicate microstructure changes^31^ and provide an indirect marker of muscle quality that is robust to device/scanner changes. We found that better muscle function was associated with values suggestive of greater image texture homogeneity (i.e., greater ASM, correlation, and IDM, lower entropy and contrast). Image entropy provided the greatest association with overall muscle function, and interestingly had the greatest association with EI, suggesting entropy may be the favored GLCM parameter when looking at muscle quality.

Healthy skeletal muscle is largely homogenous in texture, whilst pathological muscles have a greater degree of heterogeneity.^16,33,37^ Whilst further research is needed to determine the exact relationship between GLCM parameters and muscle composition, on ultrasound images greater texture homogeneity (i.e., increased ASM, IDM, correlation, plus lower entropy, and contrast values) is likely suggestive of a reduced presence of fibrous and adipose tissue in the muscles.^10^ Conversely, higher image heterogeneous may reflect adipose tissue infiltration and interstitial fibrosis in muscle.^10,31^ In scans of parotid glands, increased heterogeneous echo texture was postulated to indicate the presence of inflammatory infiltrate patches as well as the presence of fibrosis.^41^ This is supported by scans of cattle muscle, where MRI-derived intramuscular fat infiltration was associated with markers of heterogeneous texture (e.g., higher entropy).^42^ In our study, this is supported through the associations seen between these parameters (e.g., entropy) and EI, often used a marker of increased muscular fat and/or fibrotic infiltration. Cartwright et al.^43^ found a decrease in tibialis anterior and rectus femoris muscle homogeneity in 16 individuals during 14 days of ICU hospitalization (i.e., the muscle texture became more heterogeneous), whilst in patients with DMD, Hughes et al.^16^ found dystrophic muscle contained extensive “mottled” regions (i.e., areas with heterogeneous image contrast). It was postulated that this pattern of change occurred due to mild muscle breakdown and loss of the normally well-organized muscle architecture.^44^ However, it is also possible that other changes, such as inflammation or fluid retention in the subcutaneous tissue and muscle, could lead to these ultrasonographic changes.

Such infiltration and dysfunction to the muscle are likely to disrupt the ability of the muscle to contract and therefore function correctly. Watanabe et al.^25^ found that in 145 healthy participants, quadriceps IDM and ASM was reduced in older adults, whilst entropy was increased. These are indicative of heterogeneous texture changes. In support of our findings, this study also found that reductions in muscle strength (assessed by 90° isometric knee extension torque) were associated with increased entropy as well as reduced IDM and ASM (i.e., a heterogeneous image texture). Yang et al.^31^ found that in 36 older adults (mean age 73 years), texture analysis of muscle could distinguish the differences of muscle between dynapenia and non-dynapenia. In particular, a reduction in rectus femoris strength and HGS, and slower gait speed were all associated with an increased amount of “coarseness of image texture”. Interestingly, these associations were not detected by EI. No association was seen with contrast, energy, or entropy. Martínez-Payá et al.^32^ found that there were no differences in rectus femoris ASM, entropy, IDM values between those with amyotrophic lateral sclerosis (ALS) and a control group. However, the ALS muscle did possess a higher contrast (i.e., greater variation) and greater correlation. The finding of increased correlations in pathological/weak muscle in these studies conflict our finding that higher correlation values had a favorable effect on muscle function. However, the calculation and criteria of the term “correlation” in these studies may explain the differences to our study and highlight the need for consistent terminology amongst studies reporting on the use of GLCM. Indeed, the plugin used for analysis computes several of the texture parameters described originally by Haralick et al.^34^ with the only parameter computed differently being correlation, which is calculated as described by Walker et al.^45^ Nonetheless, all the associations between GLCM and muscle function we observed were relatively moderate in size. This indicates that other skeletal muscle factors likely contribute to muscle function, including but not limited to other mechanical and structural factors (e.g., tendon structure, sarcoplasmic reticulum functioning) as well as neural factors (e.g., motor unit discharge rate, synchronization of motor units).^46^ Future studies should incorporate assessment of these factors.

We found several demographic and clinical characteristics were associated with different GLCM parameters. We found that sex was predictive of correlation (greater in males) and contrast (greater in females). In 20 healthy young adults, Molinari et al.^10^ found that correlation was higher in males than females, and conversely, contrast was lower than females. This is suggestive of lower image homogeneity (as quantified by in females) and may be potentially due to a higher degree of muscle fibrous and adipose tissue present.^19,47^ We observed that high albumin associated with reduced ASM (more heterogenetic image). Albumin is often used as a crude marker of nutritional status, and it may be that increased albumin represented an increased state of muscle wasting, and likely, poor muscle quality. In contrast to the other findings, a high BMI was associated with greater correlation—a marker of similar gray-level regions and better physical function. The previous study by Yang et al.^31^ found no association with BMI and texture features of the lower limb skeletal muscles. We found that greater Hb was associated with greater ASM, IDM, and lower entropy (more homogeneity in image texture). With no physiological mechanism behind this finding, greater Hb is likely a marker of better disease control and improved overall health status. Although Watanabe et al.^25^ described that GLCM values changed with age (images become more heterogenetic), we did not observe such finding. We also observed no effect of ethnicity, although this could have been due our largely White cohort. We found no changes in GLCM values with eGFR despite including a range of CKD stages.

### Strengths and Limitations

Our cohort was an opportunistic sample of participants who had taken part in research studies in our group. These studies primarily used ultrasonography to assess skeletal muscle size but have been re-analyzed to investigate the use of GLCM as a marker of muscle function. As such, no a priori sample size was calculated, although our analysis of 90 patients is larger than many similar studies utilizing GLCM analysis on images of muscle. We analyzed scans of the rectus femoris muscle in the leg. Muscles of the lower limb are of great importance regarding their role in mobility and fall risk. Nonetheless, given the potential differences in GLCM parameters across muscles,^10,31^ further research could look at the association between these values and muscle function in different areas of the body. In particular, assessment of various synergistic muscle may offer greater insight into their role of distinctive functional tasks. Whilst our research has begun to provide evidence for the use of GLCM in the assessment of skeletal muscle, several questions remain. Data regarding tissue composition (e.g., fat infiltration, fibrosis) are required to identify the main factors influencing GLCM values. This remains an active area of interest in our group. Furthermore, the influence of muscle architecture and the ultrasound probe orientation on the texture analysis is yet to be clarified. Given the importance of improving skeletal muscle health in CKD, further study should investigate changes in GLCM parameters following interventions such as exercise or diet. It is important to state that this feature-based characterization is currently still not available in commercial scanners and requires subsequent analysis on image processing software (e.g., ImageJ). Whilst this may limit current usage, future scanners may be able to provide this form of analysis. Further research is needed to determine optimal components of GLCM analysis (e.g., bias toward transducer focal region and the role of subcutaneous fat).

## Conclusion

All GLCM parameters, a form of higher-order texture analysis, were associated with muscle function, although the largest association as seen with image entropy. Coefficients were small indicating that other properties may be influencing muscle function. Nonetheless, given the association with first-order image analysis (e.g., EI), then GLCM texture analysis may provide an indicator of muscle quality that is robust to changes in scanner settings. The use of GLCM analysis in skeletal muscle is novel and further research is needed to substantiate our findings. The sensitivity of GLCM parameters to exercise, an intervention known to improve muscle function, should be explored.

## Supplemental Material

sj-pdf-1-uix-10.1177_01617346211009788 – Supplemental material for Quantitative Muscle Ultrasonography Using 2D Textural Analysis: A Novel Approach to Assess Skeletal Muscle Structure and Quality in Chronic Kidney Disease Click here for additional data file. Supplemental material, sj-pdf-1-uix-10.1177_01617346211009788 for Quantitative Muscle Ultrasonography Using 2D Textural Analysis: A Novel Approach to Assess Skeletal Muscle Structure and Quality in Chronic Kidney Disease by Thomas J. Wilkinson, Jed Ashman, Luke A. Baker, Emma L. Watson and Alice C. Smith in Ultrasonic Imaging
