# Supplementary material for: Quantitative Muscle Ultrasonography Using 2D Textural Analysis: A Novel Approach to Assess Skeletal Muscle Structure and Quality in Chronic Kidney Disease
Source: Ultrason Imaging. 2021 Apr 15;43(3):139–48. doi: 10.1177/01617346211009788 (PMC8114433; doi:10.1177/01617346211009788)
Supplement: sj-pdf-1-uix-10.1177_01617346211009788 – Supplemental material for Quantitative Muscle Ultrasonography Using 2D Textural Analysis: A Novel Approach to Assess Skeletal Muscle Structure and Quality in Chronic Kidney Disease [file sj-pdf-1-uix-10.1177_01617346211009788.pdf]

Supplementary material 1. Test-retest differences

N=5	Test 1	Re-test	P†	ICC
Echo intensity, AU	48.8 (9.4)	46.6 (11.7)	.212	.951
ASM, AU	0.0007 (0.0002)	0.0007 (0.0002)	.580	.671
Entropy, AU	7.56 (0.14)	7.55 (0.20)	.708	.884
IDM, AU	0.14 (0.2)	0.13 (0.04)	.467	.671
Correlation, AU	0.001 (0.000)	0.002 (0.001)	.178	.277
Contrast, AU	83.4 (17.8)	112.4 (85.8)	.433	.400

Unless stated otherwise, data shown as mean (standard deviation). ASM = energy or angular second moment; IDM = homogeneity or inverse difference moment; ICC = intraclass correlation coefficient. † = significant difference assessed by paired sample t-test
